# Internet-Based Delivery of Evidence-Based Health Promotion Programs Among American Indian and Alaska Native Youth: A Case Study

**DOI:** 10.2196/resprot.6017

**Published:** 2016-11-21

**Authors:** Christine M Markham, Stephanie Craig Rushing, Cornelia Jessen, Gwenda Gorman, Jennifer Torres, William E Lambert, Alexander V Prokhorov, Leslie Miller, Kelly Allums-Featherston, Robert C Addy, Melissa F Peskin, Ross Shegog

**Affiliations:** ^1^ Department of Health Promotion and Behavioral Sciences School of Public Health University of Texas Health Science Center at Houston Houston, TX United States; ^2^ Northwest Portland Area Indian Health Board Portland, OR United States; ^3^ Alaska Native Tribal Health Consortium Anchorage, AK United States; ^4^ Inter Tribal Council of Arizona, Inc. Phoenix, AZ United States; ^5^ Oregon Health & Science University Portland, OR United States; ^6^ The University of Texas MD Anderson Cancer Center Houston, TX United States; ^7^ Rice University Houston, TX United States; ^8^ The Cooper Institute Dallas, TX United States

**Keywords:** adolescents, American Indian, Alaska Native, health promotion, Internet

## Abstract

**Background:**

American Indian and Alaska Native (AI/AN) youth face multiple health challenges compared to other racial/ethnic groups, which could potentially be ameliorated by the dissemination of evidence-based adolescent health promotion programs. Previous studies have indicated that limited trained personnel, cultural barriers, and geographic isolation may hinder the reach and implementation of evidence-based health promotion programs among AI/AN youth. Although Internet access is variable in AI/AN communities across the United States, it is swiftly and steadily improving, and it may provide a viable strategy to disseminate evidence-based health promotion programs to this underserved population.

**Objective:**

We explored the potential of using the Internet to disseminate evidence-based health promotion programs on multiple health topics to AI/AN youth living in diverse communities across 3 geographically dispersed regions of the United States. Specifically, we assessed the Internet’s potential to increase the reach and implementation of evidence-based health promotion programs for AI/AN youth, and to engage AI/AN youth.

**Methods:**

This randomized controlled trial was conducted in 25 participating sites in Alaska, Arizona, and the Pacific Northwest. Predominantly AI/AN youth, aged 12-14 years, accessed 6 evidence-based health promotion programs delivered via the Internet, which focused on sexual health, hearing loss, alcohol use, tobacco use, drug use, and nutrition and physical activity. Adult site coordinators completed computer-based education inventory surveys, connectivity and bandwidth testing to assess parameters related to program reach (computer access, connectivity, and bandwidth), and implementation logs to assess barriers to implementation (program errors and delivery issues). We assessed youths’ perceptions of program engagement via ratings on ease of use, understandability, credibility, likeability, perceived impact, and motivational appeal, using previously established measures.

**Results:**

Sites had sufficient computer access and Internet connectivity to implement the 6 programs with adequate fidelity; however, variable bandwidth (ranging from 0.24 to 93.5 megabits per second; mean 25.6) and technical issues led some sites to access programs via back-up modalities (eg, uploading the programs from a Universal Serial Bus drive). The number of youth providing engagement ratings varied by program (n=40-191; 48-60% female, 85-90% self-identified AI/AN). Across programs, youth rated the programs as easy to use (68-91%), trustworthy (61-89%), likeable (59-87%), and impactful (63-91%). Most youth understood the words in the programs (60-83%), although some needed hints to complete the programs (16-49%). Overall, 37-66% of the participants would recommend the programs to a classmate, and 62-87% found the programs enjoyable when compared to other school lessons.

**Conclusions:**

Findings demonstrate the potential of the Internet to enhance the reach and implementation of evidence-based health promotion programs, and to engage AI/AN youth. Provision of back-up modalities is recommended to address possible connectivity or technical issues. The dissemination of Internet-based health promotion programs may be a promising strategy to address health disparities for this underserved population.

**Trial Registration:**

Clinicaltrials.gov NCT01303575; https://clinicaltrials.gov/ct2/show/NCT01303575 (Archived by WebCite at http://www.webcitation.org/6m7DO4g7c)

## Introduction

As of 2012, an estimated 5.2 million individuals in the United States identified as American Indian and Alaska Native (AI/AN) alone or combined with other races, comprising 2% of the nation’s population [1]. The AI/AN population is diverse, with 567 federally recognized tribes; 60% of AI/AN individuals live in metropolitan areas and 22% live on reservations or other trust lands [1]. The AI/AN population is younger and faster growing than other US racial groups, with 30% under 18 years of age, making adolescent health a major priority [1]. Nationally, AI/AN youth are more likely to engage in sexual risk and substance use behaviors than their racial/ethnic peers [2], contributing to health disparities in teen births, sexually transmitted infections, unintentional injuries, and suicide. In 2014, AI/AN females aged 15-19 years had the third highest teen birth rate in the United States (27.3 per 1000 vs 38.0 for Hispanics, 34.9 for blacks, and 17.3 for whites) [3]; however, AI/AN females represent the highest prevalence of repeat teen births (21.6% versus 20.9% for Hispanics, 20.4% for blacks, and 14.8% for whites) [4]. Furthermore, in 2011 AI/AN females aged 15-24 years reported the highest age-specific rates of chlamydial infections among US women [5]. Between 1999 and 2009, AI/AN youth aged 15-19 were more than twice as likely as white youth to be at risk for unintentional injuries, and three times as likely to be at risk for suicide [6].

AI/AN youth also face disparities related to obesity and diabetes. Previous studies have indicated that 20-30% of AI/AN children are obese [7], compared to 17% of children nationally [8]. Furthermore, in 2001 AI/AN youth aged 10-19 years were nine times more likely to be diagnosed with type 2 diabetes compared to non-Hispanic whites (1.74 per 1000 vs 0.19 per 1000) [9]. Sequela from these health conditions often carry over into adulthood, making heart disease and cancer the leading causes of death among AI/AN adults [10]. AI/AN adults also experience higher rates of hearing loss than other racial/ethnic groups [11].

These health disparities could potentially be ameliorated by the dissemination and implementation of evidence-based adolescent health promotion programs. The United States Department of Health and Human Services’ Office of Adolescent Health, Substance Abuse and Mental Health Services Administration, and Centers for Disease Control and Prevention (CDC) have identified adolescent health promotion programs proven to impact behavior change across various health domains, including sexual health, violence and substance use prevention, physical activity, and nutrition [12-14]. However, the public health impact of health promotion programs depends both upon their efficacy and their reach [15]. To be impactful, evidence-based programs must have sufficient *reach* to their intended audience, be *implemented* as intended in the real world, and adequately *engage* their audience [16].

Limited data exist regarding the reach and implementation of evidence-based health promotion programs among AI/AN youth. The Bureau of Indian Education (BIE) supports 183 primary and secondary schools in 23 states, serving approximately 50,000 students. A school health assessment of BIE schools conducted in New Mexico in 2006 reported that 38 of 39 schools (97%) provided health education and/or health promotion activities, and 67% used a comprehensive health education curriculum [17]. Curriculum content centered predominantly on violence and substance use prevention, physical activity, and nutrition. Less than 50% of the schools provided education on pregnancy prevention, highlighting the sensitive nature of sexual health education in AI/AN communities. Ten of the 39 schools (26%) had certified health educators. No information was provided regarding whether curriculum content was evidence-based or culturally sensitive [17]; however, few culturally based or culturally sensitive behavioral health promotion programs exist for AI/AN youth [18]. Furthermore, data from the 2013 Youth Risk Behavior Surveillance Survey indicate that AI/AN students in Alaska and Montana (states with high proportions of rural and remote communities) were less likely to receive school-based human immunodeficiency virus/acquired immune deficiency syndrome education compared to white students [19]. Although restricted in scope, these findings indicate that limited trained personnel, cultural barriers, and geographic isolation may hinder the reach and implementation of evidence-based health promotion programs among AI/AN youth [1,20].

Technology-based programs may offer a viable strategy to increase the reach and implementation of evidence-based health promotion programs in this underserved population [21]. Furthermore, AI/AN youth themselves have repeatedly voiced the need for technology-based health programs to address sensitive health topics [22,23]. Although Internet access is highly variable in tribal communities across the United States, it is swiftly and steadily improving [24]. For example, BIE-funded schools are part of the federal ConnectED initiative to increase Internet connectivity and educational technology in schools. Technology usage rates among AI/AN youth exceed national averages, and many use the Internet to access health information [25,26]. These factors suggest that utilizing the Internet could increase the reach of evidence-based health promotion to AI/AN communities. Internet-based programs can also improve the implementation of programs as intended, given the reduced need for specialized facilitator training and enhanced confidentiality to deliver sensitive topics, such as sexual health [21]. Internet-based programs may also increase student engagement with program activities [21,27], and provide the ability to tailor instructions to individual characteristics (eg, gender, risk factor, or stage of change) [28]. Most importantly, Internet-based health promotion programs have been shown to impact behavior change across multiple health domains, including sexual health, substance use, physical activity and nutrition, and hearing protection [26-31].

Recently, Internet-based sexual health promotion programs have been specifically developed or adapted for AI/AN youth, with high satisfaction ratings reported [21,24]. The purpose of this study was to examine the potential of using the Internet to increase the reach and implementation of evidence-based health promotion programs across a variety of health topics, and to engage early adolescent AI/AN youth in 3 geographically dispersed regions in the United States. The findings from this study have broader implications in understanding the degree to which Internet-based programs may increase the dissemination and utilization of evidence-based health promotion programs in tribal communities.

## Methods

### Study Design

This study presents a secondary analysis of data collected during the implementation phase of a randomized controlled trial (Clinicaltrials.gov NCT01303575) that assessed the effectiveness of Native It’s Your Game (Native IYG; a Web-based sexual health education program adapted for AI/AN youth) relative to a comparison suite of 5 evidence-based Internet-based health promotion programs, across 3 geographically dispersed regions in the United States. A detailed description of the adaptation process for Native IYG is provided in a supplemental file [32]. Primary outcomes of the randomized controlled trial are forthcoming. Data presented here provide insight into the potential of using the Internet to increase the reach and implementation of evidence-based adolescent health promotion programs across a variety of health topics to AI/AN youth in diverse geographic regions, and the ability of Internet-based programs to engage AI/AN youth.

### Participants

Participants were primarily self-identified AI/AN youth aged 12-14 years and adult site coordinators (teachers, counselors, nurses, wellness coordinators, and college students) that were recruited from 25 study sites. The sites were located in 13 urban and 12 rural/tribal settings in Alaska, Arizona, and the Pacific Northwest (Oregon, Idaho, and Washington), and comprised schools, tribal community centers, after school programs, and summer youth programs. Given the importance of confidentiality when partnering with AI/AN communities, specific tribal names have been withheld. The study was approved by the Alaska Area Institutional Review Board (IRB), the Portland Area Indian Health Service IRB, the University of Texas Health Science Center at Houston (UTHealth) IRB, and 16 tribal organizations (ie, tribal councils, tribal health boards, villages, and community agencies).

### Procedure

Study activities were coordinated regionally by 3 organizations that collectively serve 295 federally recognized AI/AN tribes. Regional staff used convenience sampling to recruit tribal communities that were interested in participating in an early adolescent sexual health study. Regional staff sent flyers to schools, tribal community centers, after school programs, and summer camp programs, and advertised on organizational websites, social media outlets, and/or via newsletters. Participating sites were randomized to a treatment condition (Native IYG, n=14) or a comparison condition (n=11) featuring a suite of 5 evidence-based Internet-based health promotion programs that were not focused on sexual health.

#### Site Coordinator Training

Site coordinators at each study site completed the Collaborative Institutional Training Initiative Program’s online certification in human subject research and a live webinar (tailored for treatment or control conditions) coordinated by UTHealth research staff, which explained intervention content and protocols for logging-in youth, documentation, and maintaining confidentiality.

#### Internet-Based Health Promotion Programs

Native IYG is a 13-lesson, multimedia sexual health education curriculum for AI/AN youth (aged 12-14 years). The curriculum was adapted from an Internet-based curriculum for urban middle schools titled *It’s Your Game-Tech* (IYG-Tech) [32]. Lessons are approximately 35 minutes long. Adaption and formative development, guided by feedback from AI/AN youth and adults, comprised surface alterations (eg, changing the program logo) and deep cultural adaptations (eg, adding a blessing by AI elders and videos featuring AI/AN youth). A detailed description of the adaptation process has been published elsewhere [32].

The 5 evidence-based, Internet-based programs that comprised the comparison suite addressed hearing loss (Dangerous Decibels), alcohol use (N-Squad), tobacco use (A Smoking Prevention Interactive Experience; ASPIRE), drug use (Reconstructors), and physical activity and nutrition (The Quest to Lava Mountain). Each program has undergone usability testing with non-AI/AN youth as part of its own formative development, and has demonstrated efficacy to positively impact health behaviors and/or related psychosocial factors in other adolescent populations. The Dangerous Decibels Virtual Exhibit was developed by the Oregon Museum of Science and Industry as an online component of a public health campaign to reduce the incidence and prevalence of noise-induced hearing loss and tinnitus (ringing in the ear) by improving knowledge, attitudes, and protective behaviors of school-aged children [33]. N-Squad and Reconstructors, developed by the Rice University Center for Technology in Teaching and Learning, are Internet-based adventures for middle school students to learn about alcohol’s interaction in the digestive, circulatory, and nervous systems, and explore the science behind drugs of abuse [34,35]. ASPIRE is an online tobacco prevention and cessation curriculum, developed jointly by researchers at The University of Texas MD Anderson Cancer Center and UTHealth, with demonstrated efficacy in preventing smoking onset in high school youth [36]. The Quest to Lava Mountain, developed as part of the Texas Department of Agriculture NutriGram program by The Cooper Institute, is an educational game designed to raise awareness about healthy eating and physical activity [37].

Site coordinators logged participants onto the programs on desktop or laptop computers located in quiet locations (eg, a computer lab, empty classroom, or library). Sites with insufficient bandwidth to accommodate simultaneous Internet access by multiple users were provided with uploadable versions of their respective programs on a Universal Serial Bus (USB) drive or digital video disc (DVD).

### Data Collection

#### Assessing Reach and Implementation

Prior to implementation, site coordinators completed a computer-based education inventory survey [32] and connectivity and bandwidth testing [38] to document access parameters related to program reach. The site coordinators used problem logs during implementation to document program errors and technical issues that impacted implementation.

#### Assessing Youth Engagement

We used Likert-type scales adapted from previous studies to assess youths’ perceptions of engagement [39-42]. *Ease of use* was based on how easy it was to use the program, using a Likert-type 3-point scale (*very easy* to *kind of hard*). *Understandability* was based on whether youth understood the words used and if they needed hints from an adult to play the game, using a Likert-type 3-point scale (*yes*, *no*, and *don’t know*). *Credibility* was based on youths’ perceptions of content correctness, using a Likert-type 3-point scale (*right*, *wrong*, and *don’t know*) and trustworthiness (*yes*, *no*, and *don’t know*). *Likeability* was based on how much youth liked the program activities, using a Likert-type 5-point scale (*dislike a lot* to *like a lot*). *Perceived impact* was based on whether youth thought the program would help them make healthy decisions, using a Likert-type 3-point scale (*yes*, *no*, and *don’t know*). *Motivational appeal* was based on whether youth would play more program lessons if available, if they would recommend the program to classmates, and whether the program was *as much or more fun* than other lessons at school, including other health lessons, computer-based lessons, and their favorite video game, using a Likert-type 3-point scale (*yes*, *no*, and *don’t know*).

Ratings were collected via an Internet-based Qualtrics usability survey administered at the completion of each program. Demographic characteristics (gender, age, and self-identified race/ethnicity) were collected during the study’s baseline survey, using an Internet-based Qualtrics self-report survey [43]. Upon entering the study, youth received a unique study identification number to link data across surveys. No names were associated with the surveys. All youth provided signed parental consent and youth assent prior to participating in the study. A detailed description of study procedures has been published elsewhere [43].

#### Data Analysis

We used descriptive statistics (frequencies, median/mean, and/or range) to summarize data on reach parameters (computer access, connectivity, and bandwidth) and implementation (program errors and technical issues). Regarding youth engagement, for each parameter we calculated the percent of youth who rated each program favorably, and the range of ratings across all 6 programs, from lowest to highest.

## Results

### Reach and Implementation

Twenty-four site coordinators (24/25, 96%) provided complete or partial information related to Internet reach parameters. Eighteen computer labs, one classroom, and one after school classroom across the 3 regions were primarily composed of personal computers (13/20, 65%) and Mac computers (6/20, 30%), were mostly wired (16/18, 89%), and most had access to the Internet (22/24, 92%). Primary Web browsers included Chrome (7/20, 35%), Safari (5/20, 25%), Internet Explorer (5/20, 25%), Firefox (2/20, 10%), and Mozilla (1/20, 5%). Download speeds ranged from 0.24 megabits per second (Mbps) to 93.5 Mbps (mean 25.6 Mbps, standard deviation 31.14; median 6.37 Mbps).

At some sites, the method of program delivery changed during implementation. Treatment sites commenced the study accessing Native IYG as an online streaming program (n=12) or as an uploadable program from a USB drive (n=2). During the study, several sites that initially accessed Native IYG as an online streaming program converted to uploading Native IYG from a USB drive (n=4) due to the inability of local bandwidth to accommodate larger Native IYG video files while providing simultaneous streaming for multiple youth.

Comparison condition sites commenced the study by accessing the suite of health promotion programs via online streaming (n=8) or a combination of online streaming and an uploadable program from DVDs (n=2). Information regarding program access was missing from one site. During the study, most sites continued to access these programs via Internet connections (n=8).

The most commonly reported problems that were documented by site coordinators during implementation included frozen screens (4/6 programs), activities taking a long time to load (3/6 programs)—both of which were related to multiple simultaneous users—and trouble navigating the programs (3/6 programs).

### Youth Engagement

During implementation, 387 youth received Native IYG, of whom 191 (49%) provided feedback; 136 youth received the comparison suite of programs, of whom 108 (79%) completed at least one feedback survey. Across programs, participants were 48-60% female, with a mean age of 13.1-13.3 years, and 85-90% self-identified as AI/AN (Table 1).

**Table 1:** Demographic characteristics for AI/AN youth who provided ratings for each program (n=40-191): Alaska, Arizona, and Pacific Northwest, 2012-2014.

		Native IYG (n=191)	Dangerous Decibels (n=62)	N-Squad (n=62)	ASPIRE (n=52)	Reconstructors (n=45)	Lava Mountain (n=40)
**Gender, n (%)**						
	Female	114 (59.7)	30 (48.4)	34 (54.8)	25 (48.1)	24 (53.3)	21 (52.5)
	Male	77 (40.3)	32 (51.6)	28 (45.2)	27 (51.9)	21 (46.7)	19 (47.5)
Self-identify as AI/AN, n (%)		164 (85.9)	54 (87.1)	56 (90.3)	46 (88.5)	40 (88.9)	34 (85.0)
Mean age (standard deviation)		13.1 (0.98)	13.2 (0.83)	13.3 (0.84)	13.3 (0.77)	13.2 (0.78)	13.3 (0.88)

Youth generally rated the programs as easy to use (68-91%) and the majority understood the words in the programs (60-83%). However, some participants needed adult assistance or hints to complete the programs (16-49%). Over half of the youth reported that the program content was credible, rating it *correct* (58-90%) and *trustworthy* (61-89%), and 63-91% reported that the programs would help them make better choices. Regarding likeability, 59-87% of youth liked the programs *a little* or *a lot*. In terms of motivational appeal, 35-63% of youth reported that they would play more lessons from each program, if available; 37-66% would recommend the programs to a classmate. Although fewer youth considered the programs *as much or more fun* than their favorite video game (25-61%), youth stated that the programs were *as much or more fun* than other lessons at school (62-87%), other health lessons at school (61-79%), and other computer-based lessons at school (57-85%; Multimedia Appendix 1).

## Discussion

AI/AN youth face multiple health challenges compared to youth of other racial/ethnic groups. Viable program delivery strategies that overcome limited personnel with training in health education, cultural barriers, and geographic isolation (ie, the Internet) are needed to increase the reach and implementation fidelity of evidence-based adolescent health promotion programs in tribal communities. We examined the potential of using the Internet to deliver 6 evidence-based health promotion programs to AI/AN youth living in urban and rural settings in 3 geographically diverse regions of the United States. The health topics addressed included sexual health, hearing loss, substance use, physical activity, and nutrition. Despite variability in connectivity and bandwidth, most sites were able to access the programs via the Internet. However, technical and connectivity issues led some sites to access the programs via back-up modalities (ie, uploading the programs from a USB drive or DVD). Practitioners interested in implementing Internet-based programs in tribal communities are advised to provide contingency plans as back-ups to technical failures. We also recommend conducting bandwidth assessments, especially when dealing with multiple simultaneous users, prior to implementing an Internet-based program.

Adult site coordinators from each tribal community (including teachers, counselors, nurses, wellness coordinators, and college students) facilitated youths’ access to the Internet-based programs. Regarding implementation fidelity and program errors, site coordinators reported issues with frozen screens (4/6 programs), activities taking a long time to load (3/6 programs), and trouble navigating the programs (3/6 programs). Given that 16-49% of youth needed assistance to complete the programs, some adult oversight is recommended when implementing Internet-based programs for youth. Although the site coordinators received webinar training prior to program implementation, the training focused primarily on research study-specific protocols (eg, logging students onto computers using study identification numbers and reporting technical issues). The training related to actual program implementation, such as reviewing specific program content, was limited to approximately one hour, indicating that a broad range of personnel (including those not certified in health education) may be able to implement Internet-based health promotion programs.

Overall, AI/AN youth rated the programs favorably. Although fewer youth rated the programs as being *as engaging as their favorite video game* (a lofty expectation for any educational program), over 60% stated that the programs were *as much or more fun* than other lessons at school. These findings indicate that Internet-based health promotion programs are engaging to AI/AN youth in the diverse settings of both rural (reservation and village) and urban locations. These results also support previous studies indicating that Internet-based health promotion programs provide an engaging educational format for Millennium learners. Internet-based programs are uniquely positioned to allow for: the provision of motivational learning experiences delivered via video, animated characters, and gaming formats; the provision of role modeling activities that influence normative perceptions and skills; confidential and personalized presentation of sensitive and potentially embarrassing issues (eg, sexual health); and intervention messages that are tailored to specific user characteristics [27,28]. These features may help to engage and retain AI/AN youth in health promotion programs.

It is worth noting that youth rated Native IYG most favorably across all programs on 10 of the 13 usability parameters, possibly because this program was specifically adapted for AI/AN youth. This finding aligns with previous studies that point to the value of cultural tailoring [44]. Incorporating traditional AI/AN values and teaching methods into Internet-based health promotion programs may strengthen or reinforce a sense of cultural identity and belonging among AI/AN youth, better align with health epistemologies and learning styles, and help protect against engaging in early risk behaviors [24]. The evidence-based approach that we used to adapt IYG for AI/AN youth may provide a useful model for adapting other health promotion programs [32]. The favorable ratings for Native IYG are also noteworthy, given the sensitivity associated with sexual health education in AI/AN communities, and indicate that Internet-based programs may be an appropriate delivery channel for other sensitive health topics.

Across all programs, we experienced attrition in the number of youth who received the programs compared to those who completed a feedback survey. This attrition may have been due to respondent burden or fatigue in completing surveys after each program. However, the relatively high motivational appeal ratings across programs (eg, at least 60% of youth stated that the programs were *as much or more fun* than other lessons at school) suggest that the programs would be engaging for AI/AN youth in school, after school, or in community-based settings.

This study has several strengths. First, most feasibility studies for technology-based health promotion programs feature small numbers of participants (typically <30 users), as they do not require statistical significance to determine major usability problems [45]. In contrast, at least 40 AI/AN youth reviewed each program, with a relatively even mix of males and females in each program. Second, participants were recruited from a range of urban and rural settings across 3 geographic regions, thereby increasing the representation of youth from varied tribal communities that represent differing cultural perspectives, traditions, and values, as well as varied urbanicity and Internet access. Third, the study included Internet-based programs across a variety of health promotion topics that are important to AI/AN communities. Taken together, these factors may enhance the generalizability of findings to AI/AN communities beyond the study sample.

Despite these strengths, several limitations should be noted. First, the sample was restricted to early adolescent AI/AN youth, aged 12-14 years; thus, findings may not apply to older AI/AN youth. Second, social desirability may have biased youths’ ratings of the programs; however, the fact that some items scored lower than others (eg, comparing programs to their favorite video game) suggests that youth answered honestly, based on their experience with each program. Third, the intervention program, Native IYG, was specifically adapted for AI/AN youth. In contrast, no attempt was made to adapt the comparison group programs for AI/AN youth; this factor may have negatively impacted program ratings relative to Native IYG. Finally, although the use of percent ranges to evaluate engagement is sufficient for this kind of exploratory trial, more thorough quantitative analyses, such as appropriately powered randomized pilot tests of the programs’ impact on behavioral determinants or randomized controlled efficacy field trials, are needed to determine long-term behavioral impact.

Despite these limitations, this study demonstrates the potential of using the Internet to disseminate evidence-based health promotion programs to AI/AN youth across 3 separate geographic regions. These findings may have broader implications for understanding the degree to which Internet-based programs may enhance the reach and implementation of evidence-based health promotion programs in tribal communities, and provide an educational format that is engaging for AI/AN youth.

## Appendix

Multimedia Appendix 1

## Abbreviations

AI/AN: American Indian and Alaska Native (AI/AN)
BIE: Bureau of Indian Education
CDC: Centers for Disease Control and Prevention
DVD: digital video disc
IRB: Institutional Review Board
IYG: It’s Your Game
Mbps: megabits per second
USB: Universal Serial Bus
UTHealth: University of Texas Health Science Center at Houston
