# Supplementary material for: Internet-Based Delivery of Evidence-Based Health Promotion Programs Among American Indian and Alaska Native Youth: A Case Study
Source: JMIR Res Protoc. 2016 Nov 21;5(4):e225. doi: 10.2196/resprot.6017 (PMC5138449; doi:10.2196/resprot.6017)
Supplement: Multimedia Appendix 1 [file resprot_v5i4e225_app1.pdf]

Multimedia Appendix 1. Engagement ratings for Internet-based health promotion programs among American Indian/Alaska Native youth (n=40-191): Alaska, Arizona, and Pacific Northwest, 2012-2014.

Parameters	Native IYG (n=191)	Dangerous Decibels (n=62)	N-Squad (n=62	Aspire (n=52)	Reconstructors (n=45)	Lava Mountain (n=40)	Range of agreement across programs (%)
	Agreed (%)	Agreed (%)	Agreed (%)	Agreed (%)	Agreed (%)	Agreed (%)	
**Ease of use**							
Very easy or kind of easy	91	75	72	90	68	71	68-91
**Understandability**							
Words were understandable	83	60	70	82	77	73	60-83
Needed hints to play the game	33	28	28	16	40	49	16-49
**Credibility**							
Information was correct	90	65	64	80	58	66	58-90
Information was trustworthy	89	69	73	78	61	68	61-89
**Likeability**							
Liked a lot or a little	87	59	66	70	64	84	59-87

**Perceived impact**							
Information will help me make better choices	91	77	68	86	71	63	63-91
**Motivational appeal**							
Would play more lessons in the program if available	63	40	38	35	42	51	35-63
Would recommend to a classmate	66	41	41	37	43	61	37-66
As much or more fun than other lessons at school	82	66	62	67	74	87	62-87
As much or more fun than other health lessons at school	79	71	64	61	74	75	61-79
As much or more fun than other computer-based lessons at school	85	66	66	57	71	79	57-85
As much or more fun than favorite video game	48	35	33	25	42	61	25-61
